# Pseudoalignment tools as an efficient alternative to detect repeated transposable elements in scRNAseq data

**DOI:** 10.1093/bioinformatics/btac737

**Published:** 2022-12-15

**Authors:** Jaime Martínez de Villarreal, Mark Kalisz, Gabriel Piedrafita, Osvaldo Graña-Castro, Dafni Chondronasiou, Manuel Serrano, Francisco X Real

**Affiliations:** Epithelial Carcinogenesis Group, Spanish National Cancer Research Centre-CNIO, Madrid, Spain; CIBERONC, Madrid, Spain; Epithelial Carcinogenesis Group, Spanish National Cancer Research Centre-CNIO, Madrid, Spain; CIBERONC, Madrid, Spain; Epithelial Carcinogenesis Group, Spanish National Cancer Research Centre-CNIO, Madrid, Spain; Departamento de Bioquímica y Biología Molecular, Facultad de CC Químicas, Universidad Complutense de Madrid, Madrid, Spain; Bioinformatic Unit, Spanish National Cancer Research Centre-CNIO, Madrid, Spain; Department of Basic Medical Sciences, Institute of Applied Molecular Medicine (IMMA-Nemesio Díez), School of Medicine, San Pablo-CEU University, CEU Universities, Boadilla del Monte, Madrid, Spain; Institute for Research in Biomedicine (IRB Barcelona), Barcelona Institute of Science and Technology (BIST), Barcelona, Spain; Institute for Research in Biomedicine (IRB Barcelona), Barcelona Institute of Science and Technology (BIST), Barcelona, Spain; Epithelial Carcinogenesis Group, Spanish National Cancer Research Centre-CNIO, Madrid, Spain; CIBERONC, Madrid, Spain; Department of Medicine and Life Sciences, Universitat Pompeu Fabra, Barcelona, Spain

## Abstract

**Motivation:**

Transposable elements (TE) have played a major role in configuring the structures of mammalian genomes through evolution. In normal conditions, the expression of these elements is repressed by different epigenetic regulation mechanisms such as DNA methylation, histone modification and regulation by small RNAs. TE re-activation is associated with stemness potential acquisition, regulation of innate immunity and disease, such as cancer. However, the vast majority of current knowledge in the field is based on bulk expression studies, and very little is known on cell-type- or state-specific expression of TE-derived transcripts. Therefore, cost-efficient single-cell-resolution TE expression analytical approaches are needed.

**Results:**

We have implemented an analytical approach based on pseudoalignment to consensus sequences to incorporate TE expression information to scRNAseq data.

**Availability and implementation:**

All the data and code implemented are available as Supplementary data and in: https://github.com/jmzvillarreal/kallisto_TE_scRNAseq.

**Supplementary information:**

Supplementary data are available at *Bioinformatics* online.

## 1 Introduction

Transposable elements (TE) are heterogeneous genomic sequences that represent a large proportion of eukaryotic genomes and whose expression is related to various biological processes such as embryonic development, innate immune response and disease such as cancer (Burns, 2017; Chuong *et al.*, 2016; Deniz *et al.*, 2019). However, the repetitive nature of TE as well as their organization into families of highly similar TE members pose analytical challenges for their individual identification. It follows that most common transcript alignment procedures are oriented towards the detection of protein-coding genes and are tailored to achieve a unique mapping of reads to a reference genome locus thus ignoring multiple-site matches for being considered indicative of poor-quality reads that could incur in misalignment. This has largely precluded studies on the TE expression landscape (Bourque *et al.*, 2018).

Recently, He *et al.* designed an algorithm, referred to as scTE, capable of allocating and collapsing TE reads from single-cell RNA sequencing (scRNAseq) to TE metagenes based on the TE type-specific sequence. In an extensive analysis, they elegantly show specific TE expression in mouse embryonic stem cells (mESC), during human cardiac differentiation, mouse gastrulation and early organogenesis, in mouse adult somatic cells and in and in cells reprogrammed with OCT4, SOX2, KLF4 and MYC (OSKM) (He *et al.*, 2021). This work represents, to our knowledge, the second scRNAseq-specific TE analytical approach after Shao and Wang (2021) described an alternative strategy to quantify TE expression at single-cell resolution using transcript assembly. Both studies based TE expression quantification on the base-to-base alignment of reads using the well-established RNAseq aligner STAR (Dobin *et al.*, 2013) but allowing a certain level of multimapping due to the high degree of sequence homology among the multiple copies of different TE family members along the genomes (‘–outFilterMultmapNmax’ parameter set to 100 for He *et al.* and to 500 for Shao and Wang). Note that while the value set for this threshold becomes somehow arbitrary, it will necessarily have to compromise the detection of repeated TE with some more error-permissive mapping of individual copies. In recent years, pseudoaligners, such as Kallisto or Salmon, have appeared as an alternative and highly efficient strategy to quantify transcript abundance in bulk RNAseq data (Bray *et al.*, 2016; Patro *et al.*, 2017). More recently, pseudoalignment has been applied to scRNAseq data with a similar performance in cell-type annotation results at a much lower computational cost, an especially critical feature of single-cell genomics (Du *et al.*, 2020). Here, we leverage this principle and present an additional method to quantify TE expression at the single-cell level based on the pseudoalignment of scRNAseq reads to consensus TE sequences using Kallisto.

## 2 Implementation

To test the validity of our approach, TE consensus sequences consisting of 463 loci specific to *Mus musculus* were retrieved as a multifasta file from Repbase database (Bao *et al.*, 2015). These sequences included all repeat element classes in Repbase: DNA transposons, long terminal repeat (LTR) retrotransposons, endogenous retroviruses, non-LTR retrotransposons, simple repeats, multicopy genes and integrated viruses. Briefly, in this database TE sequences are clustered according to their similarity. The consensus sequence is generated with the 50% majority rule applied from the multiple alignments of TE copies (the number of sequences aligned depends on the size of the TE family). Each consensus sequence is defined by the most common nucleotide in that position. The resulting sequence is extended, following this method, at both sides to cover full-length TE. The termini are determined (or predicted) by the sequence identity among copies, the signatures of TE groups (e.g. terminal inverted repeats, LTR, polyA tail, Helitron’s A—TC.CTAG—T termini …), target site duplications, and the sequence similarity to known TE. Kallisto pseudoaligns reads to a reference, producing a list of transcripts that are compatible with each read while avoiding alignment of individual bases and, therefore, bypassing the multiple-mapping issues related to TE detection by conventional alignment tools. It does so by creating an index through a transcriptome de Brujin Graph where nodes are k-mers. Reads are hashed and pseudoaligned to a transcript based on their intersection of the k-compatibility classes.

**Fig. 1.**
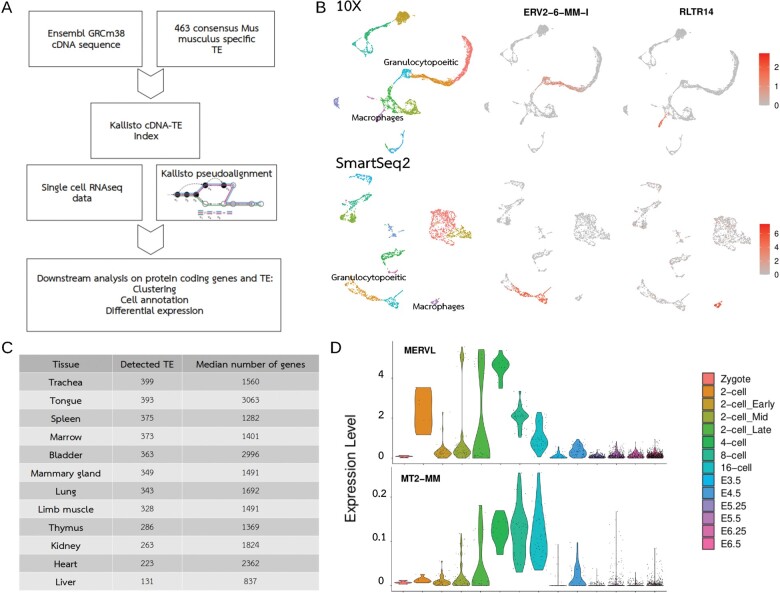
TE expression in scRNAseq data using pseudoalignment to consensus sequences. (**A**) Flowchart describing the strategy used for TE expression detection. (**B**) ERV2-6-MM-I and RLTR14 cell-specific expression in adult bone marrow in mouse both in 10× Genomics and SmartSeq2 data from Tabula Muris. (**C**) TE expression in all 10× Genomics datasets from Tabula Muris. (**D**) Expression of MERVL and MT2-MM during early mouse pre-implantation embryogenesis. In all analyses, sparse matrices were loaded into Seurat package (version 4.0.0). Seurat objects were generated for each individual sample and normalization was carried out with SCTransform function. Clusters stability was determined from different resolution values using clustree program and the final clusters annotated. Top cluster markers were determined with FindAllMarkers function. Detected TEs in the analysis were extracted from row names of the normalized Seurat object and the top TE identified from the list of cluster markers

In our study, an index was built using as a reference the combination of GRCm38 cDNA sequence assembly and the 463 consensus sequences retrieved from Repbase (Fig. 1A, Supplementary data). We were able to distinguish cell-type-specific expression of TE in granulocytopoietic (ERV2-6-MM-I) and macrophage (RLTR14) lineages from Tabula Muris data from adult murine bone marrow (Fig. 1B) (Schaum *et al.*, 2018; GSE109774). Remarkably, gene detection capacity was not hampered when compared to the same analysis without including the 463 TE consensus sequences in the aforementioned Kallisto index (Supplementary Table S1 and Supplementary Fig. S1). Importantly, the same TE expression patterns were found indistinctly using either droplet-based 3′-end 10× technology or full transcript SmartSeq2 protocol, demonstrating the applicability of this method to different single-cell-based transcriptomic technologies. TE expression was detected in all Tabula Muris 10× datasets analyzed, as shown in Figure 1C, with a moderate relationship observed between the number of TE detected and the depth of gene quantitation in each tissue. Datasets from 10× Genomics were pseudoaligned to this index using ‘Kallisto bus’ command (default parameters: k-mer length 31 bp and reads mapped to multiple genes discarded) and sparse matrices were generated from the output using bustools program and BUSspaRse R package. Datasets from SmartSeq2 protocol were pseudoaligned using ‘Kallisto pseudo’ command and sparse matrices were generated from the output (Supplementary data). Finally, in Figure 1D, we show cell-type-specific TE expression in the mouse pre-implantation embryonic dataset used in Shao and Wang (2021) (GSE45719, GSE100597, GSE109071 and E-MTAB-6967). As shown by Shao and Wang, MERVL and MT2-MM are expressed mostly during the 2–8 cell stage. Similarly, He *et al.* showed that both MERVL and MT2-MM were highly expressed in two cell-like populations arising from mESC cultures. Moreover, TE family expression distribution, as annotated in the multifasta file, showed a pronounced stage specificity (Supplementary Fig. S2).

## 3 Conclusion

In summary, we validated the use of pseudoalignment of scRNAseq data to TE consensus sequences as an alternative and cost-efficient strategy for incorporating TE expression information into the routine analysis of single-cell transcriptomic data. This approach precludes retrieving positional information on the genomic coordinates of individual TEs. However, it bypasses multiple-mapping issues, adding an additional layer of potentially valuable biological information without compromising coding gene quantification.

## Supplementary Material

btac737_Supplementary_Data
