# Supplementary material for: Pseudoalignment tools as an efficient alternative to detect repeated transposable elements in scRNAseq data
Source: Bioinformatics. 2022 Dec 15;39(1):btac737. doi: 10.1093/bioinformatics/btac737 (PMC9825736; doi:10.1093/bioinformatics/btac737)
Supplement: btac737_Supplementary_Data [file btac737_supplementary_data.pdf]

Martínez-Villarreal J, Kalisz M, Piedrafita G, Graña-Castro O, Chondronasiou D, Serrano M, Real FX.
Pseudoalignment tools as an efficient alternative to detect repeated transposable elements in scRNAseq data.

Supplementary Materials

	Kallisto report parameter	Without TE sequences	With TE sequences
**Marrow_7_2**	k-mer length	31	31
	number of targets	118489	118952
	number of k-mers	100614952	101297656
	number of equivalence classes	433624	438810
	processed reads	263061938	263061938
	pseudoaligned reads	206938412	207299844
**Marrow_7_3**	k-mer length	31	31
	number of targets	118489	118952
	number of k-mers	100614952	101297656
	number of equivalence classes	433624	438810
	processed reads	286345317	286345317
	pseudoaligned reads	226371035	226704697

Supplementary Table 1. Kallisto report parameters for the two TM 10X marrow samples in the analyses with and without including the 463 consensus TE sequences.

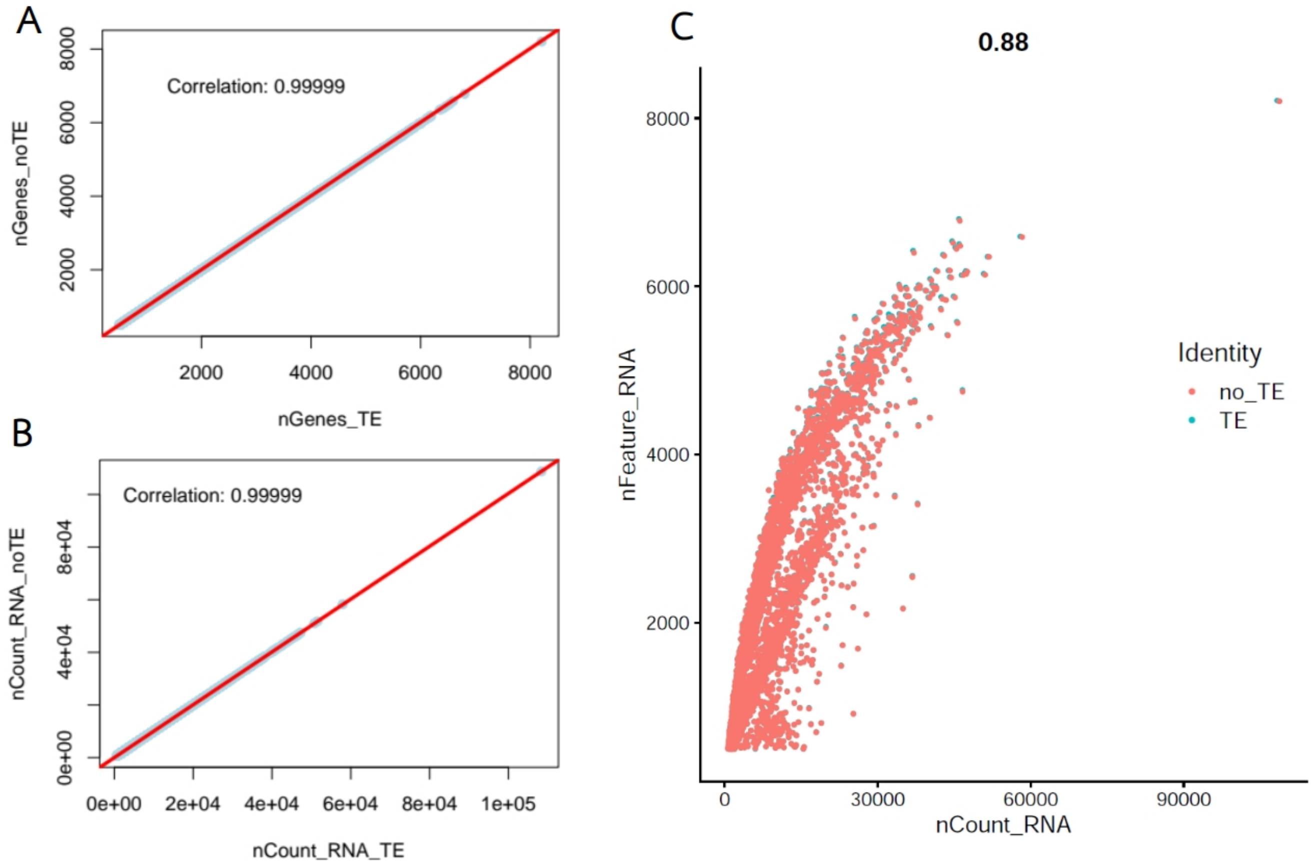

Supplementary Figure 1: **Number of genes and counts detected with and without TE sequences**. **A**. Correlation between the number of genes detected in the marrow analysis with and without the inclusion of the 463 consensus TE sequences. **B**. Correlation between the number of RNA molecules or counts with and without the inclusion of the 463 consensus TE sequences. **C.** Correlation between genes (nFeature) and counts in the merged analysis of TM 10X marrow data with and without including the 463 consensus TE sequences. An almost complete overlap of cells is observed with a slight increase of gene detection in TE (blue dots) versus non TE (red dots).

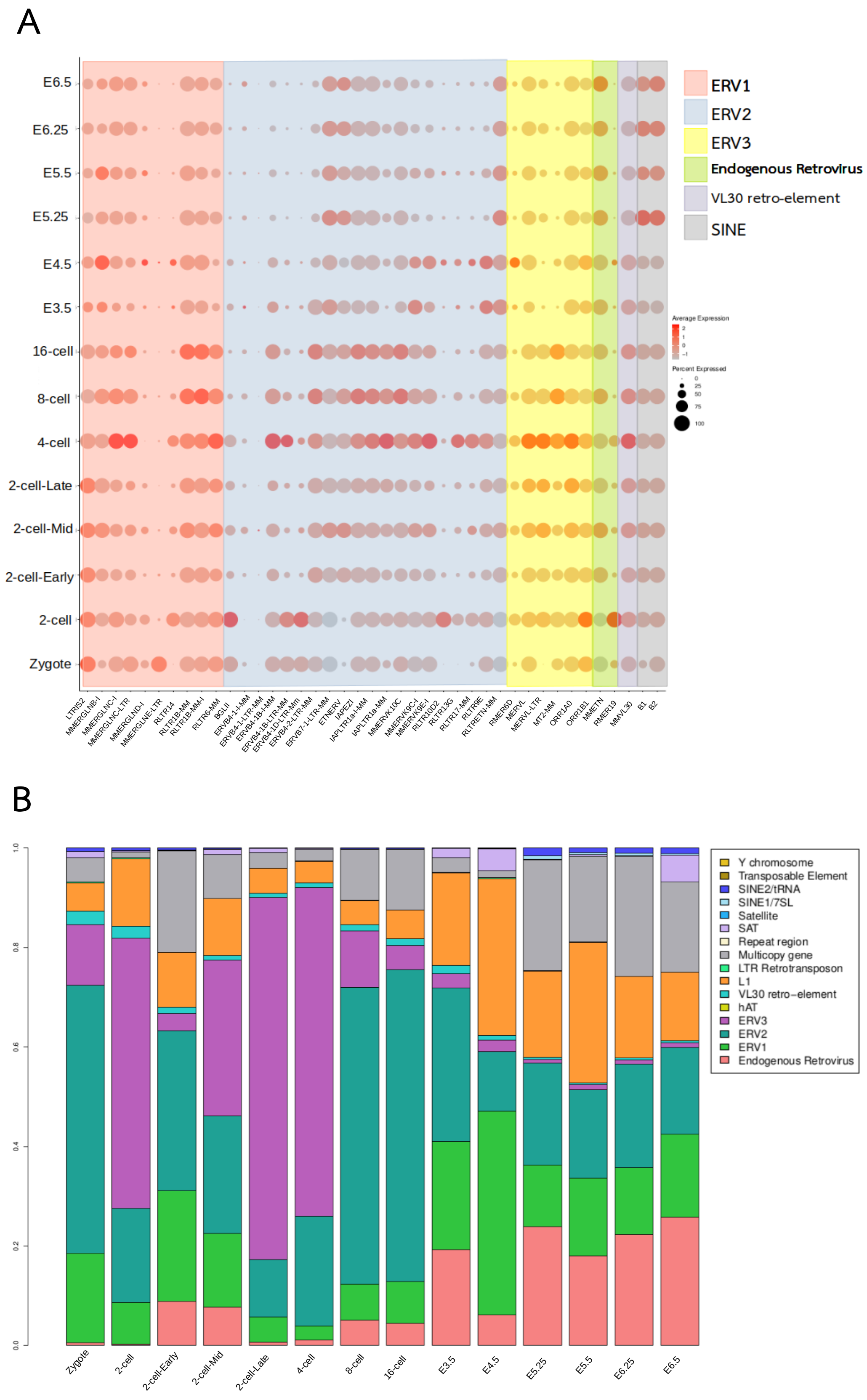

Supplementary Figure 2: **TE family expression per embryonic stage. A**. Dot plot representing TE shown in Shao and Wang Figure 3F. **B**. TE family (annotated in Repbase) counts distribution per embryonic stage.
